# Dish-Choice, a Three-Color Food Label, Improves Subjective Perceptions of Nutrition Information Among Chinese Diners Compared with a Standard Nutrition Facts Label: A Self-Controlled Survey

**DOI:** 10.3390/foods15101751

**Published:** 2026-05-15

**Authors:** Jiangyue Yu, Zhuo Sun, Shupeng Mai, Tianfeng Wu, Hui Peng, Jiahui Yao, Yaping Ren, Qi Song, Wei Lu, Zehuan Shi, Liping Shen, Wenqing Ma, Zhengyuan Wang, Jiajie Zang

**Affiliations:** 1 School of Public Health, Fudan University, Shanghai 200032, China; 23211020214@m.fudan.edu.cn; 2 Division of Health Risk Factors Monitoring and Control, Shanghai Municipal Center for Disease Control and Prevention, Shanghai 201107, China; sunzhuo@scdc.sh.cn (Z.S.); maishupeng@scdc.sh.cn (S.M.); songqi@scdc.sh.cn (Q.S.); luwei2@scdc.sh.cn (W.L.); shizehuan@scdc.sh.cn (Z.S.); shenliping@scdc.sh.cn (L.S.); mawenqing@scdc.sh.cn (W.M.); 3 Shanghai Pudong New Area Center for Disease Control and Prevention, Fudan University, Shanghai 200136, China; wtfjy.love@163.com (T.W.); renyaping0226@126.com (Y.R.); 4 The Jiading District Center for Disease Control and Prevention, Tacheng Road, 264, Jiading District, Shanghai 201800, China; penny200210108@163.com; 5 Shanghai Xuhui District Center for Disease Control and Prevention, Shanghai 200237, China; xhyingyang@126.com

**Keywords:** nutrition label, Dish-Choice, perception, socio-economic status, dietary quality

## Abstract

Background: Dining out has become increasingly prevalent in China, which is associated with higher intakes of energy, fat and sodium, elevating the risk of diet-related non-communicable diseases. However, evidence on color-coded nutrition labels for onsite prepared meals remains scarce. This study aimed to examine consumers’ perceptions of Dish-Choice, a three-color-coded onsite label, in comparison with the standard Nutrition Facts Label (NFL), to evaluate subjective perceptions of this novel label. Methods: A self-controlled trial was conducted among 3008 diners from canteens in Shanghai, with completing questionnaires twice: first on NFL perceptions, then three months later on Dish-Choice. Logistic regression and paired-sample comparison were used for analysis. Results: Compared with the NFL, Dish-Choice was associated with higher perceptual scores, with greater changes in overweight/obese, males, lower socio-economic status (SES) groups and those with poor dietary quality. Conclusions: The Dish-Choice label elicits more positive perceptual responses across multiple perceptual constructs. It is particularly well-received among vulnerable populations with lower health literacy, including men, lower-SES groups, and individuals with poor dietary habits. This supports its potential as a public health tool for on-site dining settings, though further research is needed to confirm its impact on actual food choices.

## 1. Introduction

Dining out in China has become increasingly prevalent, with the national average prevalence rising from 28.3% in 2002 to 48.3% by 2012 [1]. As a megacity in China, Shanghai’s prevalence of eating out was at 55.05% in 2012–2013 [1]; almost all the students and occupational population had at least one meal out of the home daily on average. In an investigation of 34 cities in China in 2017, the dining-out rate of urban adults in the past week was 55.6% [2]. This trend is concerning as it is associated with higher intakes of total energy, fat, oil, salt, and sugar, which are significant risk factors for diet-related non-communicable diseases (NCDs) and all-cause mortality [3]. The consumption at restaurants and company/school canteens has been linked to an increase in daily total energy intake by 140 kcal and 91 kcal, and fat intake by 6.0 g and 4.3 g, respectively [1]. There is an urgent need for measures that provide nutrition guidance to help residents choose healthier food options when eating out.

For nutrition labels to effectively change consumer behavior, they must first act on consumer perception. Labels function not merely as informational tools but also as perceptual cues that shape how consumers evaluate food quality and make dietary choices [4]. Research has consistently demonstrated that the way nutritional information is presented can significantly alter consumer attitudes and behavioral intentions [5]. According to the Theory of Planned Behavior (TPB), behavioral intentions—the most proximal determinant of actual behavior—are shaped by attitudes, subjective norms, and perceived behavioral control, all of which can be modified through label-induced perceptual changes [6]. Empirical evidence supports this pathway: nutritional label interventions have been shown to significantly improve participants’ knowledge, perceived behavioral control, behavioral intentions, and actual food choices [4]. The format and visual design of labels further modulate their perceptual impact; color-coded systems enable more efficient cognitive processing and stronger perceptual responses compared with text-based formats, thereby more effectively translating nutritional information into behavioral change [7]. Labels that are clear, credible, and intuitively designed are thus more likely to shift attitudes and build the motivational foundation necessary to translate nutritional awareness into healthier food choices [7]. The World Health Organization has identified nutrition labels as a “best-buy” measure for preventing NCDs [8].

Among all styles of nutrition labels, summarized indicator systems such as Nutri-Score are considered by the International Agency for Research on Cancer as effective tools for helping the general public choose food products of higher nutritional quality and reduce their risk of chronic diseases [9]. As a widely validated graded color-coded front-of-pack label, Nutri-Score has been shown to improve consumer understanding and the ability to identify healthier options across numerous countries and population groups [10]. However, nearly all evidence supporting Nutri-Score and similar summary labels focuses on pre-packaged retail foods, with limited application and evaluation in real-world collective catering settings.

Nutrition labels, increasingly adopted worldwide, predominantly target pre-packaged foods while paying less attention to onsite-prepared meals. To date, only a small number of studies have reported the effect of color-coded nutrition labels for onsite prepared food, including initiatives at Massachusetts General Hospital [11], healthy school canteens in New South Wales, Australia [12] and Google’s Canteen [13]. In Massachusetts General Hospital, a traffic-light labeling system demonstrated that the purchase of red-labeled items decreased from 24% to 21% while that of green-labeled items increased from 41% to 46%, with the change in purchasing remaining stable during a 24-month follow-up [11]. These findings highlight the potential of well-designed onsite nutrition labels to reshape consumer perceptions and drive sustained behavioral change in real-world food environments. Nevertheless, no large-scale real-world studies have evaluated a context-adapted three-color labeling system specifically designed for Chinese canteen environments, representing a key evidence gap compared with well-established pre-packaged labels such as Nutri-Score.

During the Shanghai Healthy Canteen Project, we developed a nutrient profiling model for on-site prepared food and a corresponding nutrition label named Dish-Choice, which is a summarized indicator system that categorizes food into three color-coded tiers. As a newly developed tool, Dish-Choice has not yet undergone systematic evaluation among local populations, leaving the attitudes and subjective perceptions towards it, as well as the sensitivity and potential for improvement among different groups, unclear. To fulfill its potential as a behavioral intervention, it is essential to first understand how it is perceived across populations with diverse characteristics.

Thus, this study seeks to investigate the perceptions and attitudes of individuals with diverse characteristics, including socioeconomic status and dietary quality, towards Dish-Choice, and to identify populations that may be more receptive to the label, establishing a basis for further research into its potential to induce behavioral changes.

It should be explicitly stated that this study focuses solely on perceptual outcomes. These outcomes include subjective understanding and ease of use, trust in label information, motivational activation induced by labels, and intention to discuss labels with others. These perceptual constructs are assessed in place of objective behavioral changes. Implications of Dish-Choice for actual dietary behaviors remain as hypotheses for future research and are not treated as confirmed outcomes in the current study.

## 2. Materials and Methods

### 2.1. Study Design

A self-controlled trial was conducted to evaluate the impact of two food label designs on consumer perceptions. All participants were exposed to both label designs—a Nutrition Facts Label (NFL) for dishes at baseline (December 2023) and the Dish-Choice label during the intervention phase (March 2024).

### 2.2. Participant Recruitment

The intervention was carried out through the Shanghai Healthy Canteen Project. From December 2023 to May 2024, 125 canteens/restaurants participated in the project, with a relatively stable diner population.

Sample size calculations were performed to ensure adequate statistical power for detecting significant differences in nutritional perceptions between the intervention and control groups. The calculations were based on the primary outcome: changes in nutritional perception scores. Using a two-sided test with a significance level (α) of 0.05 and a power (1 − β) of 0.80, the following formula was employed to determine the required sample size: n = ${\left(\frac{Z_{1-\alpha /2}+Z_{1-\beta }}{\delta /\sigma_{d}}\right)}^{2}$. δ is the expected score difference between the two phases, which is defined as 1. σ_d_ is 1.6411, which is calculated from pilot trials. To account for potential dropouts and loss to follow-up, the calculated sample size was inflated by 10%, ensuring adequate power throughout the trial. The final estimated sample size was 2907, rounded up to the nearest whole number in line with standard statistical practices for sample size determination. The canteens involved in the Shanghai Healthy Canteen Project were distributed in different administrative regions of Shanghai, enhancing the sample’s representativeness of the general Shanghai population. Diners who agreed to participate in surveys completed one questionnaire in each phase.

Participants were included in the study if they met all of the following criteria: (1) aged 18 to 75 years; (2) dined frequently at the same canteen; (3) willing to provide written or verbal informed consent; (4) able to attend regular follow-up visits.

Participants were excluded if they met any of the following criteria: (1) visual impairment, color blindness, or color weakness; (2) inability to provide informed consent; (3) inability to complete the required follow-up visits.

### 2.3. Control and Intervention

#### 2.3.1. Control

**Figure 1:**
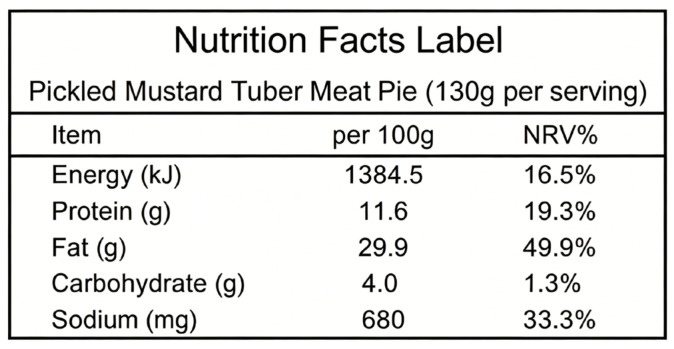
The Nutrition Facts Label (NFL).

The NFL is the most familiar nutrition information label in China. It is a complex label currently mandatory only for pre-packaged foods, not for freshly prepared foods. It presents energy and nutrients by mass (g/100 g, kJ/100 g, mg/100 g) and NRV% in tabular form (see Figure 1).

#### 2.3.2. Intervention

**Figure 2:**
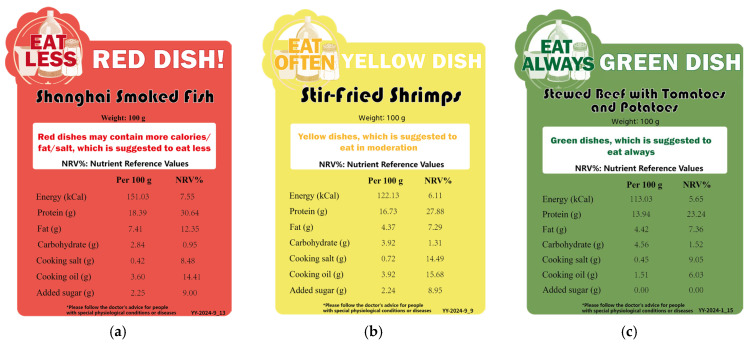
The Dish-Choice labels: (**a**) the red label; (**b**) the yellow label; (**c**) the green label.

Dish-Choice, designed by Shanghai Municipal Center for Disease Control & Prevention, is a summarized and simplified label, providing extra color classification to present the healthiness of dishes. “Red dishes” are high in nutrients that require restriction (e.g., energy, salt, oil, sugar). In contrast, “yellow dishes” and “green dishes” indicate moderate and lower levels of these nutrients, respectively. Dish-Choice also provides dietary suggestions: “red dishes” should be consumed in limited quantities, “yellow dishes” in moderation, and “green dishes” should be promoted (see Figure 2).

Among 125 canteens/restaurants, Dish-Choice was displayed for 3699 dishes, placed at windows or on bulletin boards to enhance diner awareness. During the intervention period, Dish-Choice labels for each dish were arranged horizontally by color on canteen notice boards or near the dishes for easy visibility.

Dish-Choice labels were calculated and printed based on recipes submitted by each canteen and displayed at meal selection windows of restaurants participating in the Shanghai Healthy Canteen Project. The Project lasted approximately three months, with baseline and post-intervention surveys conducted over 1–2 weeks in each restaurant, separated by an interval of about 10–11 weeks. Participants were enrolled during the baseline phase and re-investigated after the intervention.

### 2.4. Questionnaire Design

The two phases of questionnaires shared on basic personal information, eating habits, recently consumed food groups, and perceptions of nutrition labels. The difference was that the baseline questionnaire focused on perceptions of the NFL, while the intervention questionnaire focused on the Dish-Choice label.

The first section collected self-reported demographic information, including age, sex, height, weight, education, occupation, and family income. The second section used the Diet Quality Questionnaire (DQQ) [14], a validated tool jointly developed by Harvard University and Peking University, widely applied in various populations. It mainly asked participants whether they consumed specific food groups the previous day.

The third section of the questionnaire was adapted from previously established scales that have been fully validated in prior studies [15,16,17,18,19], measuring four core perceptual constructs using seven-point Likert scales: subjective understanding and ease of use were measured as the perceived fluency in reading and processing label information, and difficulty of choosing food based on label information; trust was evaluated as perceived credibility and reliability of label information; motivational activation was captured as the inspirational effect of label content; intention to discuss labels was measured as the likelihood of talking about labels with others in the near future.

Perceptual constructs were assessed by the following dimensions:

- Fluency in understanding and processing labels’ information: “How easy is it for you to read, understand, and quickly process the information on this label?” (1 = Extremely difficult to understand/process; 7 = Extremely easy to understand/process);
- Ease of choosing food: “How difficult do you feel about choosing dishes using the above nutrition labels?” (1 = Extremely difficult; 7 = Extremely easy);
- Credibility: “To what extent do you agree that this label provides reliable information?” (1 = Not credible at all; 7 = Extremely credible);
- Inspiration from the label: “To what extent do you think the information conveyed by such nutrition labels is inspiring to you (usefulness, helpfulness, etc.)?” (1 = Not inspiring at all; 7 = Extremely inspiring);
- Future social interaction about the label: “How likely are you to talk about the labels with others in the next week?” (1 = Extremely unlikely; 7 = Extremely likely).

### 2.5. Questionnaire Collection

A survey was developed for data collection and administered by trained canteen staff to better gain diners’ trust. After obtaining written consent, participants were invited to complete the questionnaire by scanning a QR code and reading questions on their own or the staff’s phone.

Municipal quality control staff organized regular training for district quality control staff. District quality controllers supervised questionnaire completion and reported progress regularly.

Given the nature of this on-site canteen-based intervention and self-reported questionnaire survey, two potential methodological biases should be noted in advance: the Hawthorne effect and social desirability bias. First, participants may alter their responses or dining-related perceptions due to the awareness of being observed and surveyed, which represents the Hawthorne effect. Second, as all outcomes relied on self-reported perceptual data, respondents might provide socially favorable and socially desirable answers rather than their real thoughts, leading to social desirability bias. Considering the repeated-measures and self-controlled study design in this research, such biases are highly relevant and may influence the consistency of subjective perception evaluation at baseline and follow-up stages.

### 2.6. Study Outcomes

The primary outcomes of the study were the proportions of participants who exhibited improvements in perceptions between the two phases. Continuous changes in perception scores were calculated as post-intervention score minus baseline score for each dimension. Positive values indicated an improvement in perception, negative values indicated a decline, and zero indicated no change. The secondary outcomes were continuous scores of the four perceptual constructs across different participant characteristics, analyzed via self-paired comparison.

### 2.7. Data Processing

Those self-reported values, such as age, weight and height, were all checked if they were validated and reasonable by researchers combining two questionnaires, in order to delete unqualified questionnaires and prepare for further data processing.

Age is divided into four levels, whose cut-offs are 30, 45, and 60, respectively. Occupation is classified according to the PRC Grand Classification of Occupation.

Socio-economic status (SES) is a composite score. First, three indicators (income, occupation and education level) were ranked and assigned scores from low to high, with an interval of 1 between adjacent levels. These scores were then summed, and SES was stratified into low (3–9 points) and high (10–12 points) [20].

Body mass index (BMI) was calculated from self-reported height and weight.

Several dietary scores with different focuses were calculated using the DQQ, including the Food Group Diversity Score (FGDS), ALL-5, NCD-Protect, NCD-Risk and Guideline-Derived Restaurant (GDR) scores:

- FGDS evaluated overall food group diversity;
- ALL-5 gauged minimal adherence to dietary guidelines, reflecting consumption across five recommended food groups [21];
- NCD-Protect (also called GDR-Healthy) reflected adherence to global dietary recommendations for healthy diet components, with higher scores indicating more health-promoting food consumption [21];
- NCD-Risk (also called GDR-Limit) reflected adherence to global dietary recommendations for components to be limited, with higher scores indicating higher consumption of restricted foods and ultra-processed foods [21];
- GDR was a comprehensive dietary score [14] including NCD-preventive dietary factors, calculated as: NCD-Protect − NCD-Risk + 9 = GDR. Higher GDR scores indicate better adherence to dietary recommendations [21].

Baseline and post-intervention scores were calculated, and the final scores used in analysis were the average of the two time points. FGDS, NCD-Protect, and GDR scores were categorized into tertiles (low, medium, high). ALL-5 and NCD-Risk were divided into two groups (low, high) according to their definitions.

### 2.8. Data Analysis

Means and standard deviations were used to describe years of dining at the restaurant, participant age, and perception levels at baseline and post-intervention. Paired-T Tests were applied to compare perception scores between the two phases.

Univariate and multivariate logistic regression models were used to assess meaningful improvements in different perception dimensions across participant characteristics. Study hypotheses were tested using a two-tailed significance level of *p* < 0.05. All analyses were conducted using SAS 9.4 for Windows (SAS Institute Inc., Cary, NC, USA).

## 3. Results

4132 individuals were investigated in the first stage, and 5130 individuals were investigated in the second stage. Among them, 3008 were included in the analysis, who completed both surveys and were confirmed eligible. This group was 22.3% males, with a mean age of 42.60 ± 14.28 years and 50.9% holding a bachelor’s degree or higher. Office clerks were the largest occupational group (24.5%), and family per capita monthly income predominantly ranged from 3500 to 7000 yuan (37.6%), followed by 7000 to 14,000 yuan (34.9%), <3500 yuan (13.5%), 14,000 to 35,000 yuan (12.1%), and ≥35,000 yuan (1.8%) (Table 1).

Paired comparisons of perception scores between the NFL and the Dish-Choice label are presented in Table 2. Across all four perceptual constructs, Dish-Choice yielded significantly higher scores than the NFL in the total sample (all *p* < 0.001). At baseline, women reported higher scores than men on all four constructs, and this pattern persisted after the intervention. Subgroup analyses by age, BMI, SES, and dietary quality indicators showed that improvements in perception scores were consistent across nearly all subgroups. The only exception was the fluency dimension among underweight participants (BMI < 18.5), where the improvement was not statistically significant (*p* = 0.05). The magnitude of the improvements, measured by Cohen’s *d*, was generally small to moderate for most perception dimensions, ranging from 0.23 to 0.50. In contrast, the effect size for future social interaction about the label was notably large, with Cohen’s *d* values exceeding 1.0 across all subgroups, indicating a substantial shift in attitudes.

In the single-factor logistic regression analysis of nine individual variables, significant predictors of perception dimensions were identified, including sex, age, BMI, SES, FGDS, and GDR (Table 3 and Appendix A Table A1).

For subjective understanding and ease of use, significant improvements were observed across multiple participant subgroups. Specifically, for fluency in understanding labels’ information, the proportion of males who benefit from using Dish-Choice was higher compared with females, with *OR* = 1.20 (1.01, 1.43) (*p* = 0.043), and the proportion of those with high SES who benefit from using Dish-Choice was smaller compared with those with low SES, with *OR* = 0.84 (0.70, 0.998) (3 decimal places kept to reflect significance) (*p* = 0.047); the proportion of those with a low FGDS who benefit from using Dish-Choice was larger compared with those with a high FGDS, with *OR* = 1.29 (1.06, 1.57) (*p* = 0.013). Regarding the ease of food choice through labels, males had an *OR* of 1.24 (1.04, 1.48) (*p* = 0.017) compared to females. Participants with a BMI from 24 to 28 had an *OR* of 1.20 (1.004, 1.42) (3 decimal places kept to reflect significance) (*p* = 0.045) compared with those with a normal BMI, and those with high SES had an *OR* of 0.81 (0.68, 0.97) (*p* = 0.020) compared to those with low SES.

For the trust in labels, the *OR* for participants aged 30 to 45 and 45 to 60 were 0.71 (0.58, 0.88) (*p* = 0.001) and 0.80 (0.64, 0.99) (*p* = 0.039), respectively, compared with those under 30 years old. Dietary quality indicators showed significant *OR*s for low FGDS, ALL-5, and GDR, with *OR*s of 1.30 (1.07, 1.57) (*p* = 0.009), 1.20 (1.03, 1.39) (*p* = 0.021), and 1.26 (1.05, 1.51) (*p* = 0.011), respectively.

The motivational activation from labels was significantly higher for participants aged 30 to 45, with an *OR* of 0.79 (0.64, 0.97) (*p* = 0.024) compared with those under 30 years old, and for those with a BMI of 28 or above, with an *OR* of 1.38 (1.02, 1.86) (*p* = 0.038) compared with those with a normal BMI. Participants with a low FGDS had an *OR* of 1.26 (1.04, 1.53) (*p* = 0.019) compared with those with a high FGDS.

The intention to discuss labels was significantly impacted only by GDR, with low and medium GDR having *OR*s of 0.66 (0.52, 0.85) (*p* = 0.001) and 0.71 (0.55, 0.92) (*p* = 0.008), respectively, compared with high GDR.

Multivariate logistic regression models were constructed to examine the associations between each dietary quality indicator (FGDS, ALL-5, GDR, etc.) and improvements in perception dimensions, adjusted for demographic characteristics including age, sex, BMI, and socioeconomic status. FGDS, ALL-5, and GDR showed statistically significant associations with all perceptual constructs (Table 4 and Appendix A Table A2). In regard to subjective understanding and ease of use, those with low dietary quality showed better improvements than those with high dietary quality. Specifically, for fluency in understanding labels’ information, compared with participants with high-level FGDS, those with low-level FGDS had an *OR* of 1.28 (1.05, 1.56) (*p* = 0.016). Compared with participants with high-level ALL-5, those with low-level ALL-5 had an *OR* of 1.19 (1.01, 1.39) (*p* = 0.032). In relation to trust in labels, compared with participants with high-level FGDS, those with low-level FGDS had an *OR* of 1.27 (1.04, 1.54) (*p* = 0.018). Compared with participants with high-level GDR, those with low-level GDR had an *OR* of 1.22 (1.02, 1.47) (*p* = 0.033). For motivational activation, compared with participants with high-level FGDS, those with low-level FGDS had an *OR* of 1.25 (1.03, 1.52) (*p* = 0.016). Regarding intention to discuss labels, the pattern was reversed compared with other perceptions: compared with participants with high-level GDR, those with low-level GDR had an *OR* of 0.65 (0.51, 0.85) (*p* < 0.05), and those with medium-level GDR had an *OR* of 0.71 (0.55, 0.91) (*p* < 0.05), both indicating significantly lower odds of positive social interaction perceptions.

## 4. Discussion

Our study demonstrates that the Dish-Choice label significantly improves four key nutritional perceptual constructs: subjective understanding and ease of use, trust, motivational activation, and intention to discuss labels. Improvements were particularly evident among males, individuals with lower SES, those who were overweight, and those with poorer dietary quality [15,22]. It is important to note that this study adopted a self-controlled (pre–post) design, where all participants were first exposed to the Nutrition Facts Label (NFL) as the pretest condition, followed by the Dish-Choice label as the posttest condition. However, unlike randomized controlled trials (RCTs) with parallel control groups, it cannot fully rule out the influence of confounding factors such as learning effects, habituation to nutrition information, or time-related changes, which may contribute to the observed improvements in perceptual constructs alongside the unique effects of the Dish-Choice label itself. These limitations are further elaborated in the limitations section below.

The Dish-Choice label outperformed the NFL in enhancing subjective understanding and ease of use. Between 30% and 40% of participants had a better perception of Dish-Choice, and 83% indicated a tendency to discuss Dish-Choice within their social communities. Consumers gravitate towards comprehensible terms and simplified color-coded information, as opposed to complex numerical data, which would necessitate a higher cognitive workload. The physical aspects of nutrition labels, including format and color contrast, significantly affect usage frequency and ease of use [22]. Standardization of these aspects may enhance the perception of nutrition labels and, consequently, the likelihood of their use. Dish-Choice’s color-coding provides a nutritional snapshot, facilitating rapid consumer judgment and improving the interpretation and application of nutrition information [23,24]. This is similar to the traffic light system, which is favored for its efficiency and preference in nutritional assessments, especially under time constraints [25]. Notably, international evidence across 12 countries confirms that summary color-coded labels such as Nutri-Score consistently achieve superior objective understanding compared with purely numeric formats [10]. Moreover, labels that combine interpretive color guidance with detailed nutrient information—consistent with the structure of Dish-Choice—can further improve subjective understanding, trust, and acceptance [26].

A previous study in America showed that individuals with greater education and higher income (broadly indicative of higher SES) were more likely to use the NFL [27], implying a relationship between the ability to process complex nutrition information and cognitive capacity, and highlighting the need for more intuitive labeling, such as a red, yellow, and green “traffic light” system [27]. Lower SES participants showed greater gains in this domain. This may be due to the complexity of the NFL widening the information-processing gap between lower and higher SES groups. Since lower-income individuals are more likely to have limited nutrition knowledge, they may find nutritional terminology confusing [28]. Dish-Choice’s summarized information provides direct dietary guidance, mitigating the need for extensive nutritional knowledge and thereby benefiting those with lower SES. This pattern aligns with evidence that Nutri-Score and similar interpretive labels disproportionately assist low-SES and low-health-literacy populations by reducing cognitive burden [10,29,30].

Individuals with poorer dietary quality exhibited larger improvements across subjective understanding and ease of use, trust, and motivational activation, likely because economic determinants of diet cost potentially mediate the relationship between SES and dietary quality. A previous investigation demonstrated that the Nutri-Score, a nutritional labeling system akin to Dish-Choice, effectively enhanced purchasing inclination towards higher-quality foods among low-income consumers, compared with the reference intake labels advocated by food manufacturers [31]. Economic factors often hinder low-income individuals from adopting healthier diets [32]. Nutritious foods tend to be more expensive, with high fruit and vegetable diets having the highest cost per calorie, while foods of lower nutritional quality typically have lower costs and are often chosen by groups with lower SES [33]. This is consistent with a systematic review [34] finding that children and adolescents from lower-SES backgrounds tend to engage in unhealthy dietary behaviors—particularly the consumption of high-energy-dense foods—while those from higher-SES backgrounds show a higher prevalence of healthful behaviors, such as increased consumption of fruits, vegetables, and dairy products, as well as greater adherence to nutritious diets. The significant perception improvement among those with poorer dietary quality suggests that Dish-Choice could substantially enhance nutritional awareness in this group, thereby reducing health disparities.

Males showed greater improvements in subjective understanding, ease of use and trust than females. Previous research has found that women are more likely to use nutrition labeling on menus [35] and generally demonstrate superior nutrition knowledge, attitudes, and behaviors compared with men [36]. Research from Poland also suggests that women pay closer attention to food labels, possibly because they more frequently shop on behalf of their families [37]. While Dish-Choice’s new format aids understanding and use among all consumers, women’s already high level of engagement with nutrition labels means that their improvement following the label change is less dramatic than that observed in men.

Age differences were observed for trust and motivational activation. Facing increased health concerns [37], middle-aged individuals show greater interest in and more positive attitudes towards nutrition labels, leading to a higher likelihood of trusting and engaging with them. In contrast, younger adults, who lack immediate health concerns, show less motivation to interact with complex nutrition information despite possessing higher levels of nutritional knowledge. Nevertheless, following the introduction of the new label, younger individuals demonstrated higher recognition and gained further nutritional knowledge through their engagement with it.

Overweight individuals benefited notably from improved subjective understanding and ease of use and motivational activation—a demographic that faces the particular challenge of maintaining a balanced diet in restaurant settings, where meals cannot be prepared under the direct guidance of nutritionists or physicians. This simplified system aids in selecting meals lower in sugar, salt, and fat, thereby enhancing nutritional decision-making and aligning with weight management goals.

Finally, higher dietary quality predicted stronger intention to discuss labels. Individuals with better dietary habits may act as voluntary advocates to spread awareness of Dish-Choice, supporting its broader adoption and public health impact.

It should be clarified that the present study only measured subjective perceptual outcomes, including perceived ease, trust, inspiration and subjective comprehension. Objective nutritional understanding and actual food choice behaviors were not evaluated. Therefore, potential behavioral and public health implications are speculative and should be considered as hypotheses for further research, rather than empirically confirmed outcomes.

### 4.1. Strengths

This study features a multicenter, community-based sample that well represents regular canteen diners, enabling a comprehensive understanding of public perceptions toward a three-color-coded labeling system. The major novelty of this work lies in its unique contextual and practical setting: it focuses on on-site prepared meals in Chinese canteens and targets the locally developed, canteen-specific Dish-Choice labeling tool, rather than merely verifying the general notion that simplified color-coded labels outperform complex formats in subjective comprehension and acceptance.

Given the absence of standardized mandatory nutrition labeling for freshly served meals in domestic catering settings, the present research adopts the context-adapted Dish-Choice intervention, providing targeted evidence for onsite food labeling practice in China. Different from previous studies conducted in simulated environments, this on-site intervention was carried out under real canteen dining conditions, which strengthens the real-world applicability of the findings.

Furthermore, this study introduces the mature simplified indicator framework of front-of-pack labeling for pre-packaged foods into dish-specific nutrition information display, offering a contextually optimized labeling strategy for collective catering.

### 4.2. Limitations

Several limitations of this study warrant consideration. First, the self-controlled design, essentially a pretest–posttest design, limits the strength of causal inference compared with randomized controlled trials. Notably, this design lacks a parallel control group that would otherwise be exposed solely to the standard Nutrition Facts Label throughout the study. All participants first received the Nutrition Facts Label and then the Dish-Choice Label, without a separate group to isolate the independent effect of the Dish-Choice Label from other confounding factors, which further weakens the causal interpretation of the results.

Second, the fixed order of label exposure, in which the Nutrition Facts Label was assessed first and the Dish-Choice Label followed, introduces inherent bias. Observed improvements in perceptions may not stem from the unique design of the Dish-Choice Label itself, but rather from learning effects, habituation to nutrition information, or other time-related factors, which will be deeply investigated in the future.

Third, the study is susceptible to the Hawthorne effect. Participants, being aware of being observed, may consciously adjust their behaviors or perceptions, which could inflate the apparent effectiveness of the intervention. In addition, social desirability bias may affect self-reported perceptions, as commonly reported in nutrition label acceptability studies, including those evaluating Nutri-Score and combined label designs [26,29]. Future studies could adopt natural experiment designs or blinded data collection methods to mitigate this bias.

Fourth, and relatedly, the study relies heavily on self-reported data rather than objective behavioral outcomes. No objective measures of dietary choices or actual changes in food consumption were collected, meaning we cannot confirm whether the observed improvements in perceived usability translate to real-world behavioral changes. Future research should incorporate objective indicators such as food purchase records or dietary intake data to strengthen the validity of the findings.

Fifth, the study may be influenced by confounding factors from broader health promotion initiatives. Conducted amid the implementation of the Shanghai Healthy Canteen Project, the research was accompanied by various health promotion activities (e.g., health education campaigns, canteen service improvements). These factors may have independently affected participants’ perceptions and behaviors, making it difficult to isolate the unique effect of the Dish-Choice Label.

Sixth, the generalizability of the findings is limited by the geographic scope of the study. Data were collected exclusively from canteens in Shanghai, where participants typically have higher levels of health literacy and socioeconomic status compared with the general population. As such, the findings may not be fully applicable to populations in less economically developed regions or those with lower health literacy.

To address these limitations, subsequent research should adopt a randomized controlled design with a parallel control group, randomize the order of label exposure, collect objective behavioral data, control for external health promotion confounding factors, and expand the geographic scope of data collection to enhance the generalizability of the findings.

## 5. Conclusions

The most obvious finding to emerge from this study is that Dish-Choice improved subjective understanding and ease of use, trust, motivational activation, and intention to discuss labels among participants with diverse characteristics compared with NFL. Among all people, those with low SES had a greater promotion of perceptions through the Dish-Choice label, which improved health equity to some extent. This study was the first research in China to evaluate the perception of the summary indicator system for dishes in real scenes. While validated color-coded labels such as Nutri-Score work well for pre-packaged food, these standardized tools are designed for Western food contexts and lack tailored frameworks for traditional Chinese dishes and on-site catering. Accordingly, Dish-Choice fills an important gap in the development and application of context-specific color-coded labeling for Chinese cuisine, delivering targeted, intuitive guidance to strengthen diners’ comprehension and acceptance. Future research should delve deeper into the purchasing behavior change through the Dish-Choice to unravel additional effectiveness, and exploration of the effect on nutrient intake change may provide valuable insights.

## Figures and Tables

**Table 1:** Individual characteristics of included diners (*n* = 3008).

Item	Content	
Age (Year), Mean ± SD		42.60 ± 14.28
Sex, *n* (%)	Male	671 (22.3)
	Female	2337 (77.7)
Dining years in this restaurant, Mean ± SD		6.41 ± 6.81
Occupation, *n* (%)	Management personnel of organs, enterprises and institutions	604 (20.1)
	Professional and technical personnel	466 (15.5)
	Office clerk	736 (24.5)
	Commercial/service personnel	381 (12.7)
	Production personnel in agriculture, forestry, animal husbandry, fishery and water conservancy	18 (0.6)
	Operator of production and transportation equipment	18 (0.6)
	Student	61 (2.0)
	Other	724 (24.1)
Education level, *n* (%)	Primary school or below	131 (4.4)
	Junior school	544 (18.1)
	Senior high school	393 (13.1)
	Junior college	409 (13.6)
	Bachelor’s degree or above	1531 (50.9)
Per capita monthly income of family (CNY), *n* (%)	<3500	406 (13.5)
	3500–7000	1132 (37.6)
	7000–14,000	1051 (34.9)
	14,000–35,000	364 (12.1)
	≥35,000	55 (1.8)

**Table 2:** Comparison between perceptions of NFL and Dish-Choice (*n* = 3008).

Perception	Variable		Promotion Rate (%)	Score (Mean ± SD)		*p*	Cohen’s *d*
				NFL	Dish-Choice		
Fluency	Total		37.2	5.51 ± 1.80	6.06 ± 1.34	***	0.383
	Sex	Male	40.57	5.26 ± 1.87	5.82 ± 1.41	***	0.379
		Female	36.24	5.59 ± 1.77	6.12 ± 1.31	***	0.384
	Age (year)	<30	37.81	5.58 ± 1.61	6.01 ± 1.29	***	0.341
		≥30, <45	35.19	5.68 ± 1.66	6.15 ± 1.24	***	0.362
		≥45, <60	37.69	5.44 ± 1.87	6.04 ± 1.37	***	0.392
		≥60	42.77	4.96 ± 2.23	5.82 ± 1.62	***	0.496
	BMI	<18.5	34.68	5.75 ± 1.65	5.97 ± 1.41	0.05	0.232
		≥18.5, <24	36.09	5.56 ± 1.76	6.05 ± 1.34	***	0.360
		≥24, <28	39.95	5.35 ± 1.91	6.08 ± 1.33	***	0.456
		≥28	40.11	5.46 ± 1.81	6.14 ± 1.20	***	0.466
	SES	Low (3–9)	38.96	5.37 ± 1.91	6.01 ± 1.42	***	0.411
		Medium (10–12)	37.49	5.45 ± 1.78	6.00 ± 1.32	***	0.394
		High (13–16)	34.83	5.74 ± 1.64	6.16 ± 1.24	***	0.336
	FGDS	Low (0–6.5)	39.27	5.35 ± 1.88	5.92 ± 1.39	***	0.383
		Medium (7–8)	37.21	5.52 ± 1.78	6.06 ± 1.32	***	0.381
		High (8.5–10)	33.48	5.79 ± 1.64	6.29 ± 1.23	***	0.386
	ALL-5	Low (0–4)	38.72	5.41 ± 1.83	5.98 ± 1.36	***	0.389
		High (4.5–5)	34.57	5.69 ± 1.73	6.19 ± 1.28	***	0.371
	NCD-Protect	Low (0–3)	39.24	5.33 ± 1.93	5.85 ± 1.42	***	0.356
		Medium (3.5–4.5)	37.83	5.44 ± 1.79	6.02 ± 1.34	***	0.406
		High (5–9)	34.91	5.74 ± 1.66	6.26 ± 1.23	***	0.386
	NCD-Risk	Low (0–1.5)	37.55	5.49 ± 1.86	6.07 ± 1.33	***	0.402
		High (2–13)	36.82	5.54 ± 1.73	6.04 ± 1.34	***	0.361
	GDR	Low (0–10.5)	38.27	5.38 ± 1.82	5.86 ± 1.39	***	0.343
		Medium (11–12)	38.42	5.39 ± 1.89	6.02 ± 1.38	***	0.410
		High (12.5–18)	34.38	5.83 ± 1.62	6.36 ± 1.14	***	0.410
Ease of Use	Total		36.77	5.53 ±1.64	5.98 ± 1.32	***	0.355
	Sex	Male	40.69	5.30 ± 1.69	5.76 ± 1.38	***	0.345
		Female	35.64	5.60 ± 1.62	6.04 ± 1.29	***	0.358
	Age (year)	<30	39.16	5.52 ± 1.50	6.00 ± 1.22	***	0.400
		≥30, <45	34.78	5.69 ± 1.52	6.08 ± 1.21	***	0.346
		≥45, <60	36.62	5.48 ± 1.68	5.92 ± 1.38	***	0.334
		≥60	41.16	5.08 ± 2.08	5.73 ± 1.63	***	0.418
	BMI	<18.5	32.88	5.73 ± 1.49	5.96 ± 1.31	*	0.248
		≥18.5, <24	35.5	5.56 ± 1.62	5.98 ± 1.32	***	0.338
		≥24, <28	39.68	5.44 ± 1.70	5.98 ± 1.33	***	0.394
		≥28	42.25	5.38 ± 1.69	6.01 ± 1.24	***	0.476
	SES	Low (3–9)	38.54	5.44 ± 1.75	5.91 ± 1.41	***	0.351
		Medium (10–12)	37.84	5.42 ± 1.65	5.94 ± 1.27	***	0.416
		High (13–16)	33.71	5.75 ± 1.47	6.09 ± 1.23	***	0.309
	FGDS	Low (0–6.5)	38.77	5.36 ± 1.72	5.83 ± 1.37	***	0.356
		Medium (7–8)	35.7	5.54 ± 1.62	5.98 ± 1.31	***	0.354
		High (8.5–10)	34.96	5.83 ± 1.46	6.24 ± 1.18	***	0.354
	ALL-5	Low (0–4)	37.62	5.42 ± 1.67	5.87 ± 1.37	***	0.347
		High (4.5–5)	35.3	5.72 ± 1.56	6.18 ± 1.18	***	0.370
	NCD-Protect	Low (0–3)	37.73	5.32 ± 1.79	5.76 ± 1.40	***	0.331
		Medium (3.5–4.5)	37.53	5.47 ± 1.63	5.93 ± 1.34	***	0.372
		High (5–9)	35.28	5.77 ± 1.48	6.21 ± 1.18	***	0.363
	NCD-Risk	Low (0–1.5)	37.1	5.50 ± 1.71	5.99 ± 1.32	***	0.373
		High (2–13)	36.4	5.57 ± 1.55	5.97 ± 1.31	***	0.333
	GDR	Low (0–10.5)	37.05	5.41 ± 1.66	5.80 ± 1.36	***	0.314
		Medium (11–12)	38.11	5.41 ± 1.70	5.92 ± 1.36	***	0.381
		High (12.5–18)	34.84	5.84 ± 1.49	6.30 ± 1.13	***	0.383
Credibility	Total		40.46	5.48 ± 1.56	6.01 ± 1.27	***	0.425
	Sex	Male	42.92	5.31 ± 1.60	5.79 ± 1.32	***	0.375
		Female	39.75	5.53 ± 1.55	6.08 ± 1.25	***	0.441
	Age (year)	<30	46.07	5.47 ± 1.41	6.05 ± 1.17	***	0.485
		≥30, <45	37.84	5.62 ± 1.48	6.09 ± 1.20	***	0.409
		≥45, <60	40.47	5.43 ± 1.57	5.98 ± 1.27	***	0.422
		≥60	41.48	5.10 ± 1.97	5.72 ± 1.61	***	0.417
	BMI	<18.5	37.84	5.61 ± 1.42	5.91 ± 1.37	***	0.314
		≥18.5, <24	40.21	5.50 ± 1.55	6.03 ± 1.24	***	0.425
		≥24, <28	41.15	5.42 ± 1.62	6.00 ± 1.30	***	0.439
		≥28	43.32	5.35 ± 1.61	6.00 ± 1.26	***	0.495
	SES	Low (3–9)	40.97	5.40 ± 1.66	5.96 ± 1.35	***	0.417
		Medium (10–12)	40.48	5.41 ± 1.57	5.98 ± 1.22	***	0.462
		High (13–16)	39.82	5.63 ± 1.41	6.10 ± 1.20	***	0.408
	FGDS	Low (0–6.5)	43.41	5.27 ± 1.65	5.91 ± 1.30	***	0.457
		Medium (7–8)	39.25	5.52 ± 1.52	6.01 ± 1.28	***	0.412
		High (8.5–10)	37.19	5.78 ± 1.40	6.22 ± 1.17	***	0.388
	ALL-5	Low (0–4)	42.03	5.38 ± 1.58	5.94 ± 1.31	***	0.436
		High (4.5–5)	37.75	5.66 ± 1.51	6.15 ± 1.18	***	0.406
	NCD-Protect	Low (0–3)	42.27	5.24 ± 1.70	5.83 ± 1.34	***	0.431
		Medium (3.5–4.5)	41.28	5.43 ± 1.54	5.99 ± 1.27	***	0.452
		High (5–9)	38.2	5.73 ± 1.41	6.19 ± 1.17	***	0.397
	NCD-Risk	Low (0–1.5)	40.01	5.45 ± 1.64	6.01 ± 1.27	***	0.429
		High (2–13)	40.95	5.51 ± 1.47	6.02 ± 1.27	***	0.421
	GDR	Low (0–10.5)	42.63	5.32 ± 1.57	5.87 ± 1.30	***	0.428
		Medium (11–12)	40.92	5.37 ± 1.63	5.95 ± 1.33	***	0.436
		High (12.5–18)	37.04	5.82 ± 1.42	6.29 ± 1.09	***	0.410
Inspiration	Total		39.49	5.43 ±1.59	5.95 ± 1.33	***	0.406
	Sex	Male	40.69	5.23 ± 1.65	5.72 ± 1.37	***	0.369
		Female	39.15	5.49 ± 1.57	6.02 ± 1.31	***	0.418
	Age (year)	<30	43.38	5.40 ± 1.47	5.95 ± 1.25	***	0.449
		≥30, <45	37.6	5.56 ± 1.52	6.02 ± 1.29	***	0.404
		≥45, <60	39.51	5.42 ± 1.59	5.92 ± 1.31	***	0.493
		≥60	40.51	5.04 ± 2.00	5.75 ± 1.60	***	0.462
	BMI	<18.5	40.54	5.49 ± 1.53	5.79 ± 1.45	**	0.290
		≥18.5, <24	38.2	5.46 ± 1.59	5.98 ± 1.30	***	0.408
		≥24, <28	40.75	5.38 ± 1.62	5.92 ± 1.38	***	0.399
		≥28	45.99	5.28 ± 1.64	6.01 ± 1.21	***	0.564
	SES	Low (3–9)	40.72	5.36 ± 1.68	5.90 ± 1.40	***	0.401
		Medium (10–12)	37.84	5.37 ± 1.60	5.92 ± 1.28	***	0.446
		High (13–16)	39.41	5.58 ± 1.46	6.03 ± 1.28	***	0.380
	FGDS	Low (0–6.5)	42.42	5.23 ± 1.66	5.83 ± 1.35	***	0.448
		Medium (7–8)	37.92	5.46 ± 1.58	5.93 ± 1.34	***	0.378
		High (8.5–10)	36.89	5.75 ± 1.43	6.18 ± 1.25	***	0.375
	ALL-5	Low (0–4)	40.82	5.31 ± 1.62	5.85 ± 1.37	***	0.414
		High (4.5–5)	37.21	5.64 ± 1.52	6.12 ± 1.24	***	0.392
	NCD-Protect	Low (0–3)	41.84	5.19 ± 1.70	5.75 ± 1.37	***	0.425
		Medium (3.5–4.5)	39.55	5.37 ± 1.60	5.91 ± 1.34	***	0.422
		High (5–9)	37.47	5.70 ± 1.45	6.15 ± 1.25	***	0.374
	NCD-Risk	Low (0–1.5)	39.06	5.42 ± 1.66	5.97 ± 1.32	***	0.422
		High (2–13)	39.97	5.45 ± 1.52	5.93 ± 1.34	***	0.388
	GDR	Low (0–10.5)	41.24	5.26 ± 1.60	5.77 ± 1.35	***	0.399
		Medium (11–12)	39.52	5.35 ± 1.66	5.89 ± 1.40	***	0.399
		High (12.5–18)	37.15	5.75 ± 1.47	6.27 ± 1.14	***	0.425
Social Interaction	Total		83.24	3.65 ± 1.24	5.59 ± 1.55	***	1.176
	Sex	Male	81.97	3.56 ± 1.27	5.43 ± 1.51	***	1.114
		Female	83.61	3.67 ± 1.23	5.63 ± 1.55	***	1.195
	Age (year)	<30	84.26	3.66 ± 1.12	5.63 ± 1.51	***	1.205
		≥30, <45	82.21	3.72 ± 1.20	5.62 ± 1.55	***	1.167
		≥45, <60	84.05	3.60 ± 1.26	5.53 ± 1.53	***	1.182
		≥60	83.28	3.51 ± 1.46	5.55 ± 1.63	***	1.152
	BMI	<18.5	81.53	3.49 ± 1.26	5.43 ± 1.65	***	1.165
		≥18.5, <24	83.12	3.70 ± 1.22	5.60 ± 1.55	***	1.157
		≥24, <28	83.22	3.60 ± 1.27	5.59 ± 1.53	***	1.192
		≥28	86.63	3.55 ± 1.23	5.59 ± 1.41	***	1.326
	SES	Low (3–9)	83.12	3.59 ± 1.29	5.56 ± 1.55	***	1.153
		Medium (10–12)	85.63	3.62 ± 1.23	5.58 ± 1.52	***	1.254
		High (13–16)	81.36	3.74 ± 1.17	5.62 ± 1.55	***	1.145
	FGDS	Low (0–6.5)	83.1	3.48 ± 1.27	5.46 ± 1.59	***	1.169
		Medium (7–8)	83.3	3.67 ± 1.26	5.58 ± 1.53	***	1.168
		High (8.5–10)	83.41	3.92 ± 1.09	5.82 ± 1.46	***	1.206
	ALL-5	Low (0–4)	82.95	3.54 ± 1.26	5.48 ± 1.59	***	1.154
		High (4.5–5)	83.76	3.84 ± 1.17	5.77 ± 1.45	***	1.220
	NCD-Protect	Low (0–3)	81.62	3.48 ± 1.30	5.37 ± 1.60	***	1.134
		Medium (3.5–4.5)	83.47	3.57 ± 1.25	5.50 ± 1.59	***	1.120
		High (5–9)	84.41	3.86 ± 1.14	5.84 ± 1.42	***	1.273
	NCD-Risk	Low (0–1.5)	84.2	3.64 ± 1.29	5.61 ± 1.55	***	1.204
		High (2–13)	82.19	3.66 ± 1.18	5.56 ± 1.54	***	1.146
	GDR	Low (0–10.5)	81.34	3.52 ± 1.23	5.38 ± 1.58	***	1.105
		Medium (11–12)	82.35	3.59 ± 1.28	5.52 ± 1.59	***	1.151
		High (12.5–18)	86.81	3.89 ± 1.16	5.94 ± 1.39	***	1.316

Statistical methods: Paired-T Test; ***: *p* < 0.001, **: *p* < 0.01, *: *p* < 0.05; Fluency and Ease of Use jointly represent subjective understanding and ease of use. Credibility represents trust. Inspiration represents motivational activation. Social Interaction represents the intention to discuss labels.

**Table 3:** Significant predictors of improved perceptions of the Dish-Choice label, based on single-factor logistic regression (*n* = 3008).

Perception	Significant Predictors (*p* < 0.05)	*OR* (95%*CI*)
Fluency	Male (vs. Female)	1.20 (1.01, 1.43)
	Low SES (vs. High SES)	0.84 (0.70, 0.998)
	Low FGDS (vs. High FGDS)	1.29 (1.06, 1.57)
	Low ALL-5 (vs. High ALL-5)	1.20 (1.02, 1.40)
Ease of Use	Male (vs. Female)	1.24 (1.04, 1.48)
	≥24, <28 BMI (vs. ≥18.5, <24)	1.20 (1.004, 1.42)
	Low SES (vs. High SES)	0.81 (0.68, 0.97)
Credibility	<30 Age (vs. ≥60)	0.71 (0.58, 0.88)
	≥30, <45 Age (vs. ≥60)	0.80 (0.64, 0.99)
	Low FGDS (vs. High FGDS)	1.30 (1.07, 1.57)
	Low ALL-5 (vs. High ALL-5)	1.20 (1.03, 1.39)
	Low GDR (vs. High GDR)	1.26 (1.05, 1.51)
	Medium GDR (vs. High GDR)	1.18 (0.98, 1.42)
Inspiration	≥30, <45 Age (vs. ≥60)	0.79 (0.64, 0.97)
	≥24, <28 BMI (vs. ≥18.5, <24)	1.38 (1.02, 1.86)
	Low FGDS (vs. High FGDS)	1.26 (1.04, 1.53)
Social Interaction	Low GDR (vs. High GDR)	0.66 (0.52, 0.85)
	Medium GDR (vs. High GDR)	0.71 (0.55, 0.92)

Note: *OR* = odds ratio; *CI* = confidence interval. Fluency and Ease of Use jointly represent subjective understanding and ease of use. Credibility represents trust. Inspiration represents motivational activation. Social Interaction represents the intention to discuss labels.

**Table 4:** Significant predictors of improved perceptions of the Dish-Choice label, based on multivariate logistic regression (*n* = 3008).

Perception	Significant Predictors (*p* < 0.05)	*OR* (95%*CI*)
Fluency	Low FGDS (vs. High FGDS)	1.28 (1.05, 1.56)
	Low ALL-5 (vs. High ALL-5)	1.19 (1.01, 1.39)
Credibility	Low FGDS (vs. High FGDS)	1.27 (1.042, 1.54)
	Low GDR (vs. High GDR)	1.22 (1.016, 1.47)
Inspiration	Low FGDS (vs. High FGDS)	1.25 (1.03, 1.52)
Social Interaction	Low GDR (vs. High GDR)	0.65 (0.51, 0.85)
	Medium GDR (vs. High GDR)	0.71 (0.55, 0.91)

Note: Multivariate logistic regression models were adjusted for age, sex, BMI, and socioeconomic status (SES). *OR* = odds ratio; *CI* = confidence interval. Fluency and Ease of Use jointly represent subjective understanding and ease of use. Credibility represents trust. Inspiration represents motivational activation. Social Interaction represents the intention to discuss labels.

## Data Availability

The original contributions presented in this study are included in the article. Further inquiries can be directed to the corresponding authors.

## Abbreviations

The following abbreviations are used in this manuscript:

SES	socio-economic status
FGDS	food group diversity scores
NCDs	non-communicable diseases
FOPL	front-of-pack nutrition labeling
NHSC	National Healthy School Canteens Project of Australia
DQQ	Diet Quality Questionnaire
NFL	Nutrition Facts Label
GDR	guideline-derived restaurant scores
BMI	body mass index
OR	odds ratio
CI	confidence interval
CNY	Chinese Yuan

## Appendix A

**Table A1:** Full single-factor logistic regression results for differences in perceptions between the NFL and Dish-Choice label (*n* = 3008).

Variable	*n* (%)	Fluency			Ease of Use			Credibility			Inspiration			Social Interaction	
		*OR* (95%*CI*)	*p*		*OR* (95%*CI*)	*p*		*OR* (95%*CI*)	*p*		*OR* (95%*CI*)	*p*		*OR* (95%*CI*)	*p*
Sex															
Male	671 (22.3)	1.199 (1.006, 1.430)	0.043		1.238 (1.039, 1.476)	0.017		1.140 (0.958, 1.356)	0.141		1.066 (0.895, 1.270)	0.473		0.891 (0.711, 1.116)	0.315
Female (ref)	2337 (77.7)														
Age (year)															
<30 (ref)	521 (17.3)														
≥30, <45	1242 (41.3)	0.893 (0.722, 1.104)	0.295		0.829 (0.671, 1.024)	0.081		0.713 (0.580, 0.877)	0.001		0.787 (0.639, 0.968)	0.024		0.863 (0.654, 1.138)	0.297
≥45, <60	934 (31.1)	0.995 (0.797, 1.241)	0.963		0.898 (0.720, 1.119)	0.338		0.796 (0.641, 0.988)	0.039		0.852 (0.686, 1.059)	0.15		0.984 (0.734, 1.320)	0.915
≥60	311 (10.3)	1.229 (0.923, 1.636)	0.158		1.087 (0.816, 1.447)	0.568		0.830 (0.625, 1.102)	0.198		0.889 (0.669, 1.182)	0.419		0.930 (0.636, 1.360)	0.71
BMI															
<18.5	222 (7.4)	0.940 (0.702, 1.259)	0.68		0.890 (0.662, 1.197)	0.441		0.905 (0.680, 1.206)	0.496		1.103 (0.830 1.465)	0.499		0.897 (0.626 1.286)	0.553
≥18.5, <24 (ref)	1848 (61.4)														
≥24, <28	751 (25.0)	1.178 (0.990, 1.402)	0.066		1.195 (1.004, 1.423)	0.045		1.040 (0.875, 1.235)	0.658		1.112 (0.935 1.323)	0.228		1.008 (0.803 1.264)	0.948
≥28	187 (6.2)	1.186 (0.872, 1.612)	0.278		1.329 (0.979, 1.804)	0.068		1.136 (0.839, 1.540)	0.409		1.377 (1.018 1.864)	0.038		1.316 (0.849 2.040)	0.22
SES															
Low (3–9) (ref)	1191 (39.6)														
Medium (10–12)	835 (27.8)	0.939 (0.783, 1.127)	0.502		0.971 (0.809, 1.165)	0.752		0.980 (0.818, 1.173)	0.823		0.886 (0.739, 1.063)	0.192		1.210 (0.946, 1.547)	0.129
High (13–16)	982 (32.6)	0.837 (0.703, 0.998)	0.047		0.811 (0.680, 0.967)	0.020		0.953 (0.802, 1.132)	0.585		0.947 (0.797, 1.125)	0.534		0.886 (0.711, 1.106)	0.285
FGDS															
Low (0–6.5)	1207 (40.1)	1.285 (1.055, 1.565)	0.013		1.178 (0.968, 1.433)	0.101		1.296 (1.068, 1.572)	0.009		1.260 (1.039, 1.530)	0.019		0.978 (0.760, 1.259)	0.864
Medium (7–8)	1126 (37.4)	1.177 (0.964, 1.439)	0.11		1.033 (0.846, 1.261)	0.751		1.092 (0.897, 1.329)	0.382		1.045 (0.858, 1.273)	0.661		0.993 (0.768, 1.282)	0.955
High (8.5–10) (ref)	675 (22.4)														
ALL-5															
Low (0–4)	1906 (63.3)	1.196 (1.024, 1.396)	0.024		1.105 (0.947, 1.290)	0.204		1.195 (1.027, 1.392)	0.021		1.164 (0.999, 1.356)	0.051		0.943 (0.773, 1.152)	0.568
High (4.5–5) (ref)	1102 (36.6)														
NCD-Protect															
Low (0–3)	925 (30.7)	1.062 (0.883, 1.276)	0.526		1.009 (0.838, 1.214)	0.927		1.042 (0.868, 1.249)	0.66		1.099 (0.916, 1.320)	0.31		0.880 (0.694, 1.114)	0.288
Medium (3.5–4.5)	986 (32.7)	0.882 (0.737, 1.054)	0.167		0.907 (0.759, 1.085)	0.287		0.879 (0.737, 1.048)	0.151		0.916 (0.767, 1.093)	0.328		1.073 (0.849, 1.356)	0.558
High (5–9) (ref)	1097 (36.4)														
NCD-Risk															
Low (0–1.5)	1582 (52.5)	0.969 (0.836, 1.124)	0.679		1.031 (0.889, 1.196)	0.687		0.962 (0.831, 1.113)	0.6		0.963 (0.832, 1.115)	0.611		1.155 (0.954, 1.398)	0.141
High (2–13) (ref)	1426 (47.5)														
GDR															
Low (0–10.5)	1147 (38.1)	1.184 (0.985, 1.423)	0.073		1.101 (0.916, 1.324)	0.306		1.263 (1.054, 1.514)	0.011		1.187 (0.990, 1.423)	0.064		0.663 (0.518, 0.848)	0.001
Medium (11–12)	997 (33.1)	1.191 (0.985, 1.440)	0.071		1.152 (0.953, 1.392)	0.144		1.178 (0.977, 1.420)	0.087		1.105 (0.916, 1.333)	0.295		0.709 (0.549, 0.916)	0.008
High (12.5–18) (ref)	864 (28.7)														

Note: *OR* = odds ratio; *CI* = confidence interval. Fluency and Ease of Use jointly represent subjective understanding and ease of use. Credibility represents trust. Inspiration represents motivational activation. Social Interaction represents the intention to discuss labels.

**Table A2:** Full multivariate logistic regression results for differences in perceptions between the NFL and Dish-Choice label (*n* = 3008).

Variable	*n* (%)	Fluency			Ease of Use			Credibility			Inspiration			Social Interaction	
		*OR* (95%*CI*)	*p*		*OR* (95%*CI*)	*p*		*OR* (95%*CI*)	*p*		*OR* (95%*CI*)	*p*		*OR* (95%*CI*)	*p*
FGDS															
Low (0–6.5)	1207 (40.1)	1.277 (1.046, 1.559)	0.016		1.147 (0.941, 1.399)	0.175		1.267 (1.042, 1.540)	0.018		1.246 (1.025, 1.515)	0.028		0.961 (0.745, 1.240)	0.76
Medium (7–8)	1126 (37.4)	1.179 (0.964, 1.442)	0.108		1.029 (0.842, 1.258)	0.78		1.082 (0.888, 1.317)	0.436		1.037 (0.850, 1.263)	0.722		0.982 (0.759, 1.269)	0.888
High (8.5–10) (ref)	675 (22.4)														
ALL-5															
Low (0–4)	1906 (63.3)	1.186 (1.014, 1.387)	0.032		1.081 (0.925, 1.264)	0.329		1.164 (0.998, 1.357)	0.053		1.143 (0.979, 1.333)	0.09		0.932 (0.762, 1.141)	0.496
High (4.5–5) (ref)	1102 (36.6)														
NCD-Protect															
Low (0–3)	925 (30.7)	1.171 (0.975, 1.407)	0.091		1.063 (0.885, 1.278)	0.513		1.147 (0.957, 1.374)	0.137		1.174 (0.980, 1.407)	0.082		0.807 (0.637, 1.021)	0.074
Medium (3.5–4.5)	986 (32.7)	1.130 (0.945, 1.353)	0.181		1.090 (0.911, 1.305)	0.346		1.125 (0.943, 1.343)	0.19		1.087 (0.910, 1.298)	0.359		0.922 (0.729, 1.167)	0.502
High (5–9) (ref)	1097 (36.4)														
NCD-Risk															
Low (0–1.5)	1582 (52.5)	0.987 (0.847, 1.151)	0.868		0.997 (0.855, 1.163)	0.969		0.962 (0.827, 1.119)	0.611		0.959 (0.824, 1.117)	0.592		1.150 (0.943, 1.402)	0.168
High (2–13) (ref)	1426 (47.5)														
GDR															
Low (0–10.5)	1147 (38.1)	1.198 (0.992, 1.447)	0.061		1.083 (0.897, 1.309)	0.406		1.224 (1.016, 1.473)	0.033		1.163 (0.966, 1.401)	0.111		0.654 (0.509, 0.842)	0.001
Medium (11–12)	997 (33.1)	1.190 (0.983, 1.439)	0.074		1.139 (0.941, 1.378)	0.181		1.165 (0.965, 1.405)	0.112		1.094 (0.906, 1.320)	0.351		0.705 (0.545, 0.911)	0.008
High (12.5–18) (ref)	864 (28.7)														

Note: Multivariate logistic regression models were adjusted for age, sex, BMI, and socioeconomic status (SES). *OR* = odds ratio; *CI* = confidence interval. Fluency and Ease of Use jointly represent subjective understanding and ease of use. Credibility represents trust. Inspiration represents motivational activation. Social Interaction represents the intention to discuss labels.
